# New England Dental Society

**Published:** 1889-02

**Authors:** Edgar O. Kinsman

**Affiliations:** Secretary; 15 Brattle Sq., Cambridge, Mass.


To the EDITOR:

At the annual meeting of the New England Dental Society held in Boston, November 15th and 16th, the following were elected officers for the ensuing year:

President, Dr. C. A. Brackett, Newport, R. I.; Vice-Presidents, Dr. C. W. Clement, Manchester, N. H.; Dr. W. E. Page, Boston, Mass.; Secretary, Dr. E. O. Kinsman, Cambridge, Mass.; Assistant Secretary, Dr. W. P. Cooke, Boston, Mass.; Treasurer, Dr. G. A. Gerry, Lowell, Mass.; Librarian, Dr. A. H. Gilson, Boston, Mass.; Executive Committee, Dr. A. M. Dudley, Salem, Mass.; Dr. L. Rideout, Lynn, Mass.; Dr. E. B. Davis, Concord, N. H.

EDGAR O. KINSMAN, D.D.S., *Secretary.*

15 Brattle Sq., Cambridge, Mass.

